# Stereotypic Behavior in Sows Is Related to Emotionality Changes in the Offspring

**DOI:** 10.3389/fvets.2020.00079

**Published:** 2020-03-12

**Authors:** Patricia Tatemoto, Thiago Bernardino, Beatrice Morrone, Mariana Ramos Queiroz, Adroaldo José Zanella

**Affiliations:** Department of Veterinary Medicine and Animal Health, Center for Comparative Studies in Sustainability, Health and Welfare, School of Veterinary Medicine and Animal Science, FMVZ, University of São Paulo, São Paulo, Brazil

**Keywords:** emotionality, fear tests, gestation, piglets, prenatal, stereotypic behavior

## Abstract

Some effects of expressing stereotypic behavior have not yet been elucidated. During gestation, the environment has the potential to interfere with offspring development and to have prenatal or longer-term consequences. We tested the hypothesis that the occurrence of stereotypic behavior during gestation could affect the phenotype of the offspring. Twenty-eight pregnant sows were studied by comparing two groups differing in the amount of stereotypy shown. We analyzed emotionality in the offspring from sows showing high or low stereotypy frequency using the open field and novel object tests. In the open field test, piglets from sows with a high rate of stereotypies walked more in central sectors (*p* < 0.0001) and lateral sectors (*p* = 0.04) than piglets from sows with a low rate of stereotypies. In the novel object test, the offspring from low stereotypy sows vocalized more (*p* = 0.008). We demonstrate for the first time that the stereotypic behavior by the mother during gestation changes the phenotype of the offspring, in particular, their emotionality.

## Introduction

Stereotypic behavior, or stereotypy, is repetitive and apparently functionless and often develops in suboptimal environments that could cause poor welfare (1). This behavior develops in animals kept in environments with few stimuli, physical restraint, fear, or frustration (1). In farm animals, this behavior can be widely observed. The environment of farmed animals does provide for some of their needs. However, the environment may be inadequate to fulfill all needs, or the animal may be unable to cope effectively with the environment. The expression of stereotypic behavior suggests frustration of highly motivated behaviors, components of which may occur even in the absence of the appropriate stimulus. However, the expression varies among individuals kept in common environments. Stereotypic behavior has been described in a wide range of species kept in an artificial environment. Furthermore, the stereotypic behavior expression is often considered a welfare indicator (1–3), since it can tell us about psychological states that are difficult to evaluate. However, this specific behavioral indicator does not correlate well with the hypothalamic-pituitary-adrenal (HPA) axis activity (4), which is mainly an indicator of short-term welfare problems.

In order to use stereotypies as an animal welfare indicator, it is necessary to understand the causal factors of its expression. Frustration, associated with the motivation to show a behavioral or physiological change that is blocked in some way, is one of the most consistent causal factors. Furthermore, the kind of stereotypy can be associated with a specific frustration. For example, frustration related to food restriction can trigger oral stereotypies in sows (5). There is also a genetic component (6) and a personality predisposition (7, 8) for expressing stereotypies in different species. In this sense, there is a synergic effect of internal and external variables that triggers this strategy for coping with problems. Some variables have more impact in triggering the stereotypy than others if we consider a range of environmental factors (9).

What is the consequence of stereotypic behavior expression in the long term for the animals? Male mink kept in artificial environments that showed more stereotypy had lower success in copulation (10). However, when animals were developing in environmentally enriched conditions, the environment could have affected the ontogeny, physiology of stress, physiological reproductive mechanisms, social behavior, and flexibility (10), increasing the difference from animals kept in a barren environment and showing stereotypies.

Considering the long-term effects of stereotypy expression by the mother, how does it change the phenotype of the offspring? In mammals, pregnancy has an important role in shaping the organism. The mother's environment may have effects on the offspring. This concept comes from the “thrifty phenotype hypothesis,” in which the neurodevelopment reprogramming induces alterations to cope with the initial environment, anticipating postnatal environment (11). In other words, the prenatal environment has the potential to adjust the offspring phenotype and prepare individuals for the environment into which they will be inserted so that they are prepared to cope better with the challenges. The environment in which an animal is maintained during gestation may result in changes in several offspring qualities (12–17). By this mechanism, factors, such as emotional reactivity, responsiveness to stressors, and cognition can be modulated by challenges in the prenatal and neonatal periods (16, 18, 19). Studies have shown that some stressors, such as negative interactions with the handler (16, 17, 20) and social stress (16) have altered emotional reactivity, social behavior, and responsiveness to stressors, cognition and memory in the offspring. Moreover, sows experiencing less hunger during gestation have offspring with reduced aggressive behavior (21).

One of the mechanisms that can affect the emotionality of the offspring during development is the maternal excess of glucocorticoids, which can affect important brain structures and generate negative effects (16, 17, 20). Glucocorticoids are important stress hormones in adult animals but also have other functions in both adults and fetuses. Fetal effects are completely different depending on gestational age, severity, and duration of the exposure (22). The effects of prenatal stress on brain structures, such as the hippocampus and amygdala may generate changes in offspring's emotionality (23, 24). To assess the emotionality of non-human animals, some tests have been validated and discussed (25). These include the open field test and the novel object test (25–28), in which behaviors, such as activity and vocalization can be used as measures of emotionality (27), which can indicate the levels of fear and exploratory motivation of each animal. Our goal was to evaluate the consequences of stereotypies during gestation for the emotionality of the offspring. As far as we know, this is the first approach relating stereotypic behavior in the mother during gestation to the phenotype of the offspring.

## Materials and Methods

### Animals

The study was conducted at the production unit of the Faculty of Veterinary Medicine and Animal Science, University of São Paulo, at the Fernando Costa's Campus, in Pirassununga. This study was approved by the Ethics Committee on Animal Use of the Faculty of Veterinary Medicine and Animal Science, University of São Paulo (CEUA/FMVZ—protocol number 3606300114).

Twenty-eight pregnant gilts were used, from a group of 36 animals, nulliparous TopGen Afrodite® (Farm Araporanga—Juaguariaíva–PR). A parallel experiment involving the same animals evaluated the effect of dietary fiber during gestation on their welfare and on the offspring. The animals were distributed by weight into two treatments (diets with high fiber, *N* = 16; or low fiber, *N* = 12). Since there was no difference (*p* > 0.05) regarding the dietary treatment on the stereotypic behavior, we assessed this behavior expression in their offspring emotionality. The sows were inseminated with pooled semen with an average age of 291 days.

Sows were group-housed in pens, with nine animals per pen, with individual feeders and water provided *ad libitum*. Immediately prior to feeding, there was an auditory signal to reduce anticipatory response related to food and to the presence of humans. During the feeding time, animals were confined in individual crates. During feeding, sows had no access to water for 20 min due to the fact that the feeders were connected, and water would mix different types of food. Thirty minutes after the beginning of feeding, all the animals were released. After being released, it was possible to enter back into the stalls since the drinker was in the same place that was kept open allowing the access to water. They were fed twice daily, at 08:00 and 15:00 h. The pen was 6.7 m wide × 4.4 m long, measuring 29.48 m^2^ (3.27 m^2^ per animal), disregarding the area of the feeders. The total area per animal consisted of 4.38 m^2^. The floor of the pens was solid concrete and covered by 12 rubber mats with 100 cm^2^ and 30 mm high (EBV 30—Vedovati®).

### Experimental Design

To assess the effects of stereotypic behavior (sham-chewing) throughout pregnancy in the offspring, the sows were ranked from high to low rate of stereotypies (*N* = 28 sows, split in two groups *N* = 14); 14 sows showed a low rate of stereotypic behavior (range of 1.49 to 17.22 s) and 14 sows showed a high rate (range of 19.77 to 71.11 s). The emotionality of piglets was analyzed individually, based on the behavioral results of the open field and novel object tests of eight piglets per sow (four couples). Principal component analysis (PCA) was performed in order to guarantee that there was no effect of the kind of diet on the data considering stereotypic behavior. In other words, the PCA was used to verify which of the assessed variables explained more of the variance in the data, ensuring that the different diets in the parallel experiment were not affecting the groups.

### Behavioral Data

In order to collect behavioral data, an ethogram was adapted from Zonderland et al. [(29), Table 1]. Behavioral measures of sows were obtained by direct observation on gestational days 29, 30, 31, 59, 60, 61, 74, 75, 76, 89, 90, and 91, consisting of the average gestational age for the sows kept in the same pen in each period. The behavioral assessments were performed by direct observation of two different time periods, 1 h before and 1 h after feeding, totaling four periods of observation each day. Five observers previously standardized the behavioral data collection to avoid bias in data collection. The behavioral data used in the analysis consisted of the collection of the behaviors' frequency performed by the focal animal with continuous observation. At each time of observation, each animal was observed three times per uninterrupted 120 s, totaling 6 min per animal per observation time (before and after feeding), with a total of 24 min per observation day. The data collection consisted of four sets, which were conducted over 3 consecutive days each, to avoid possible interference of stressful events (e.g., 29, 30, and 31 in the evaluation of the first third of pregnancy, following the same protocol on two consecutive pregnancy periods).

**Table 1:** Definition of behaviors for behavioral observation of pregnant sows.

**Behaviors**	**Definition**
Sleep	Animal sleeping
Lying ventrally	Lying with the belly on the ground with all the limbs under the body
Lying laterally	Lying sideways, with all the limbs extended laterally
Standing	Body supported by the four limbs
Sham chewing	Continuous chewing without the presence of visible food in the oral cavity
Rooting the floor	Snout touches the ground followed by head movements
Licking the floor	The tongue touches the floor and is followed by movements with the head
Interacting fence or gate	Biting or nibbling the fence wire or gate
Interacting with mats	Snout or tongue touches mats followed by head movements
Bites (E)	Bite on any parts of the body (tail, vulva, ear, body)
Facing (E)	Face to face, with a fixed view to the other animal
Pushing (E)	Pushing another animal using the head or the muzzle
Vocalization (E)	Sound emission emitted by the animal

*The caption E indicates behaviors in which only the events and not the duration were measured*.

### Farrowing

Parturition happened in individual pens, measuring 4.3 × 2.0 m each, with available material for nest building (one package of hay and sugarcane bagasse to cover the concrete floor) and iron bars, around the perimeter of the pens, in order to optimize the protection of piglets against crushing. Pens had a creep feeder made of concrete, measuring 0.97 × 2.2 m each, where the piglets had access to solid food from birth. The creep feeder also had a bed composed of dehydrated sugarcane bagasse, and the heat source was a 60-W incandescent lamp. Every farrowing was monitored using video cameras, with access via the Internet, followed by direct observation after the onset of farrowing (the videos were monitored every hour until the beginning of each farrowing). Interventions were performed only when necessary, following a pre-established protocol, allowing standardization of management procedures. The assistance consisted of palpation when the interval between births exceeded 1 h, and administration of injectable synthetic oxytocin when an absence of contractions observed exceeded 1 h (Placentex®−2 ml intramuscular). Piglets had their teeth ground and ears notched with local anesthesia in order to minimize pain, as it was a standard operating procedure at the farm. Iron administration, ear notching, and individual weighing were all performed when piglets were 1 day old. The males were not castrated and the tails were left intact.

### Weaning and Fear Tests in Piglets

Piglets were weaned at 28 days of age. At weaning, they were kept in four suspended pens; each pen consisted of four animals: two pairs from each sow (two sows per pen), totaling eight piglets per sow being used in post-weaning studies divided into four pens. Piglets had access to food and water *ad libitum*. Each animal was individually identified using a marker of non-toxic and non-permanent ink. The observer who collected and registered the piglet behavior was not aware of the treatment of the mother sows.

Tests for assessing emotionality were conducted by a combination of open field and novel object test (30, 31), which can indicate the levels of fear and exploratory motivation of each animal. The tests were performed at 30 days of age, with piglets of four pens being tested on the same day (8 piglets, of 4 pens, totaling 32 piglets tested per day). Piglets were tested one by one, returning them to their home pen, being removed one by one sequentially from the pens, so that the absence of an individual of the group remained balanced over time. The combination of tests allowed prior habituation of piglets to the test arena, wherein the open field test preceded the novel object test. The test arena (2.37 × 4.85 m) contained soil demarcations forming 48 sectors throughout the pen. Each test (open field and novel object test) lasted 5 min totaling 10 min per pig. Each piglet was gently placed in a predetermined location in the test arena and recorded during the test period. The behaviors that were quantified were latency to walk, the number of central and lateral sectors crossed, time in activity (walking), time in freezing, and vocalizations (events). After this test, a novel object was inserted (empty polypropylene yellow bucket) by a pulley system in the center of the arena. Subsequent behaviors were recorded for 5 min. In this test, we evaluated the latency to walk, time close to the object (in the sectors surrounding the object), time exploring the object (near to the object with the head turned to it), time in freezing, and vocalization (events). Although in our study vocalizations were not analyzed with appropriate bioacoustical equipment, the vocalizations found were short low-intensity ones, which are completely different to the long and loud ones (usually observed during painful procedures, such as castration). After each animal had been tested, the pen was washed with water to reduce and remove possible chemical cues and feces and urine of the animals. The pen was also washed before the first test in order to standardize the entries of the piglets in the wet test arena.

### Data Analysis

Behavioral measures were grouped in order to create categories for the sows, as follows: inactivity (sleeping, lying ventral, and lying laterally); foraging (licking the floor, rooting empty feeder); physical environment interaction (rooting floor, interacting with the fence and the gate, interacting with rubber mats); stereotypies (sham-chewing). A principal component analysis (PCA) was performed within these categories for verifying which behavior explained more the variance at each principal component (PC). The PCA was used to ensure that different diets in the parallel experiment were not affecting the groups. To perform the PCA, a singular-value decomposition of the sows behavior data was used in the software R Core Team (32), and eigenvalues higher than 1 was used as a cutoff point for retaining PCs. Based on this, the category “stereotypic behavior” was selected, and the effect of its variation in the emotionality of the piglets was analyzed. The 28 sows were split into two groups of 14 sows to understand the effect of the stereotypic behavior expression on piglets' emotionality as an indicator of animal welfare. Splitting the sows into two groups as a gradient of stereotypies expression ensures that environmental variables are standardized in all individuals. The software IBM SPSS Statistics for Windows (version 22.0) was used to compare both groups of piglets from sows with a low or high stereotypy rate with a *t*-test or Mann–Whitney *U*-test, depending on data normality assessed with the Kolmogorov–Smirnov test. The α value established for significant results was 0.05 (*p* < 0.05).

## Results

**Figure 1:**
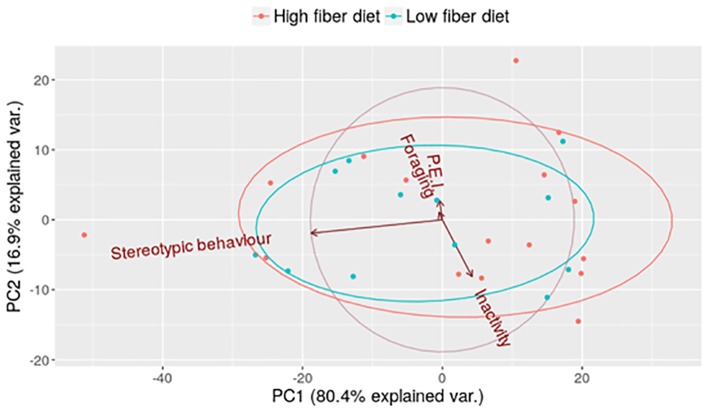
Principal component analysis. PCA evaluated in relation to behavioral categories (*N* = 28 pregnant sows group-housed; P.E.I. corresponds to physical environmental interaction).

The PCA shows 97.3% of the variation within the first two principal components, being 80.4% of the explained variation in principal component 1 (PC1) and 16.9% at PC2 (Figure 1). “Stereotypies” was the variable that most explained individual variation, 99.7% of the variation in PC1 and PC2 (Table 2). As seen in Figure 1 the points correspond to the PC1 and PC2 scores of each sow, and the ellipses indicate 68% of the sows in each fiber diet. One ellipse is inside the other indicating that the low fiber diet and the high fiber diet did not influence the behaviors.

**Table 2:** The explanation proportion of the behaviors in PC1 and PC2 variations.

**Variables**	**PC1 (%)**	**PC2 (%)**	**Total**
Inactivity	4.9	84.2	89.1
Foraging	0.1	1.5	1.6
Physical environmental interaction	0.0	9.6	9.6
Stereotypic behavior	95.0	4.7	99.7

**Figure 2:**
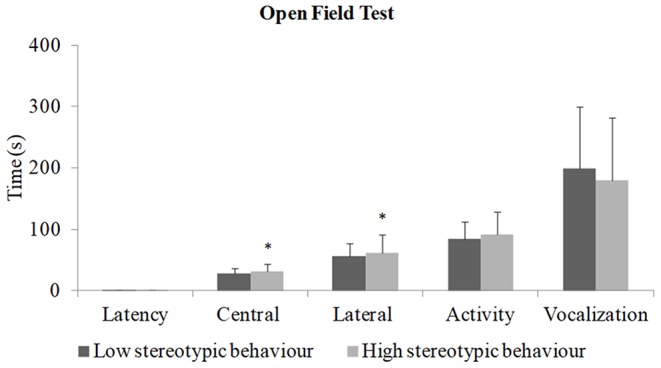
Open field test in the piglets (*N* total = 142, *N* = 76 piglets from sows with a low rate of stereotypy and *N* = 66 piglets from sows with a high rate of stereotypy). *Indicates difference in central sectors (*t*-test; *p* = 0.000) and lateral sectors (*t*-test; *p* = 0.04). There was no difference in the latency (Mann–Whitney *U*-test; *p* = 1.00), activity (*t*-test; *p* = 0.54), and vocalization (*t*-test; *p* = 0.34).

Piglets from sows with a high rate of stereotypies walked more over the sectors in relation to piglets from sows with a low rate of stereotypies as showed in the open-field test (Figure 2) with a difference in the number of central sectors (*p* = 0.000) and lateral sectors (*p* = 0.04). In both tests, no piglets showed freezing behavior.

**Figure 3:**
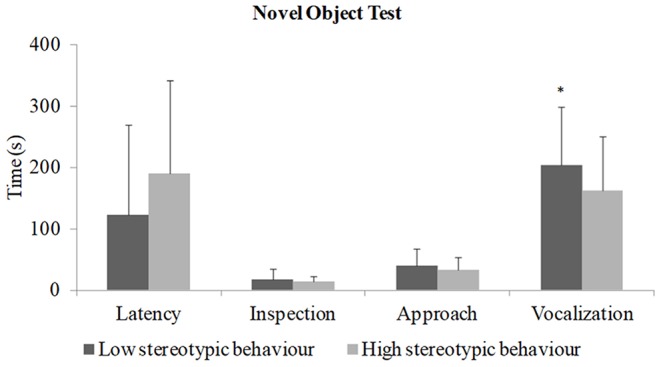
Novel object test (*N* total = 142; *N* = 76 piglets from sows with a low rate of stereotypy and *N* = 66 piglets from sows with a high rate of stereotypy) *Indicates difference on vocalization (*t*-test; *p* = 0.008). There is no difference on latency (Mann–Whitney *U*-test; *p* = 0.17), inspection (Mann–Whitney *U*-test; *p* = 0.10; *Z* = 1.61), and approach (*T*-test; *p* = 0.08).

In the novel object test (Figure 3), piglets from sows with a low rate of stereotypies vocalized more (*p* = 0.008) than piglets from sows with a high rate of stereotypies. The other variables did not differ significantly.

## Discussion

In this study, we showed that there is a relationship between stereotypies expressed by the sow and emotionality in their offspring. As far as we know, this is the first evidence that stereotypies expressed by the mother during gestation can affect the offspring emotionality, changing their phenotype. We demonstrated that stereotypies represented a factor that explained the individual variation in response to situations of challenge in the sows, such as barren environments. This may be due to different strategies that animals use to cope with the monotony and reduced complexity of the environment, common factors in commercial production environments, plus the fact that individuals may require different levels of environmental stimulation (33). In monotonous environments, the low complexity may not meet the biological needs of some animals, and they may develop stereotypies, while others may direct their motivation to the environment (physical environment interaction).

In the open field test, we have shown that piglets from sows with a high rate of stereotypic behavior walked more in the central and lateral sectors. This increased movement in the test could be an indicator of more encouraged and explorative animals, showing a potential benefit for the offspring from sows with a high rate of stereotypies when gestation occurred in challenging environments. This result agrees with another study comparing the offspring of sham-chewing to non-sham-chewing sows (34). We demonstrated in the same behavioral assessment tests that in the open field test, piglets born from non-sham-chewing sows demonstrated more latency to move in the arena and less activity, indicating more fear (34). An alternative explanation could be a response to increased anxiety in relation to novel social isolation.

In the novel object test, we showed that piglets from sows with a low rate of stereotypies vocalized more. In pigs, it has been argued that the vocalization is strongly associated with excitement levels (35). Thus, the vocalization is considered a useful tool for assessing the welfare of an individual (36), but it should be used carefully. Apparently, the low-intensity vocalizations are used to maintain social contact, while durable and high-intensity vocalizations are more related to individual mental states (37). Vocalizations are considered indicative of stressful situations for piglets. In some studies, handling situations considered clearly stressful were used to clarify what each vocalization means for the welfare of an animal. For example, studies with social isolation (38, 39), castration (40), and weaning (41) reported high rates of high-frequency vocalization (>1 kHz) when the piglets were challenged. Although in our study vocalizations were not analyzed with appropriate bioacoustical equipment, recordings were similar to the short, low-intensity vocalizations described in situations of social isolation and used as an attempt to maintain social contact with conspecifics.

The types of vocalization, which may include grunting or screams, are the result of different situations and contexts (35). Some studies suggest that high frequency and long-lasting vocalizations indicate more severe stress, but this has not been validated with physiological indicators. Few studies in swine have analyzed the relationship between vocalizations and physiological responses, such as the release of stress hormones, for example. It was demonstrated that endocrine responses (e.g., the release of adrenaline) can be accompanied by different types of vocalization (39), indicating a response of common origin in the central nervous system (35). In this way, we can understand that vocalizations may indicate emotional states of an individual. Social isolation was the biggest stressor in both tests due to the high social motivation of the pig, which can be increased by separation anxiety. Nonetheless, behavioral responses can be considered indicative of emotionality but not direct measurements.

Both tests have been used as an indicator of the animals' emotionality. While there are clear definitions of fear and anxiety, both emotions have a close relationship and can easily be confused. Fear is defined as a reaction to the actual perception of danger, while anxiety is defined as a reaction to a potential hazard (42), and in terms of evolution, emotions are highly adaptive. In the case of fear, individuals can be prepared with a cascade of physiological and behavioral responses to deal with danger, while anxiety can be considered as a previous step to prepare for the potential loss of individual's integrity (43, 44). From the evolutionary point of view, these reactions promote fitness and can increase life expectancy since both emotions modulate animal behavior to prevent exposure to risk. It also enables the individual to optimize the ability to assess the costs and benefits of certain exposures, which is related to emotional learning, one of the functions of the amygdala (45). Although these emotions have been strategically selected in the course of evolution, when in excess, it can lead to chronic stress and to a difficult adjustment of the individual to the environment, therefore reducing its welfare (25). In addition, excessive fear and anxiety can disrupt the expression of a range of desirable behaviors and reduce productivity outcomes in animal production systems (25). Fear can be related to the biology of the species, especially for prey species. Effects can be made by genetic components, previous experiences, ontogenetic factors, and the prenatal and neonatal environments.

We propose that further studies are needed to elucidate the relationship between stereotypies expressed by the mother during pregnancy and the emotionality and welfare of the offspring. Not all the elements showed by stereotypies in relation to the welfare of an animal and its offspring are clear, although there are many studies elucidating issues about this indicator. In another study (34), it is shown that maternal sham-chewing expressions are related to less fear in their offspring. However, we do not know whether the differences in the emotionality of the offspring are a consequence of the genetic (6) or personality (7, 8) predisposition associated with stereotypies in the sows. Nonetheless, this new information helps to build knowledge about the consequences of stereotypic behavior for animal welfare. Since this behavior can indicate frustration, an affective state, it is helpful to understand the consequences in the offspring.

## Data Availability Statement

The raw data supporting the conclusions of this article will be made available by the authors, without undue reservation, to any qualified researcher.

## Ethics Statement

This study was approved by the Ethics Committee on Animal Use of the Faculty of Veterinary Medicine and Animal Science, University of São Paulo (CEUA/FMVZ—protocol number 3606300114).

## Author Contributions

PT and AZ contributed to the conceptualization and writing of the original draft. PT and MQ performed the formal analysis. AZ contributed to funding acquisition, resources, and supervision. PT, TB, and BM performed the investigation. AZ, PT, and TB contributed to the methodology. PT, TB, BM, and AZ supervised the project administration. PT, TB, BM, MQ, and AZ contributed to writing, reviewing and editing the manuscript.

### Conflict of Interest

The authors declare that the research was conducted in the absence of any commercial or financial relationships that could be construed as a potential conflict of interest.
